# Choroid plexus dysfunction impairs beta-amyloid clearance in a triple transgenic mouse model of Alzheimer’s disease

**DOI:** 10.3389/fncel.2015.00017

**Published:** 2015-02-06

**Authors:** Ibrahim González-Marrero, Lydia Giménez-Llort, Conrad E. Johanson, Emilia María Carmona-Calero, Leandro Castañeyra-Ruiz, José Miguel Brito-Armas, Agustín Castañeyra-Perdomo, Rafael Castro-Fuentes

**Affiliations:** ^1^Department of Human Anatomy, School of Medicine, University of La LagunaTenerife, Spain; ^2^Institute of Neurosciences and Department of Psychiatry and Forensic Medicine, Autonomous University of BarcelonaBarcelona, Spain; ^3^Department of Neurosurgery, Alpert Medical School at Brown UniversityProvidence, Rhode Island, USA; ^4^Department of Physiology, School of Medicine, University of La LagunaTenerife, Spain

**Keywords:** Alzheimer disease, 3xTg-AD mice, choroid plexus, dysfunction, amyloid-β, collagen-IV, transthyretin, aquaporin-1

## Abstract

Compromised secretory function of choroid plexus (CP) and defective cerebrospinal fluid (CSF) production, along with accumulation of beta-amyloid (Aβ) peptides at the blood-CSF barrier (BCSFB), contribute to complications of Alzheimer’s disease (AD). The AD triple transgenic mouse model (3xTg-AD) at 16 month-old mimics critical hallmarks of the human disease: β-amyloid (Aβ) plaques and neurofibrillary tangles (NFT) with a temporal- and regional- specific profile. Currently, little is known about transport and metabolic responses by CP to the disrupted homeostasis of CNS Aβ in AD. This study analyzed the effects of highly-expressed AD-linked human transgenes (APP, PS1 and tau) on lateral ventricle CP function. Confocal imaging and immunohistochemistry revealed an increase only of Aβ42 isoform in epithelial cytosol and in stroma surrounding choroidal capillaries; this buildup may reflect insufficient clearance transport from CSF to blood. Still, there was increased expression, presumably compensatory, of the choroidal Aβ transporters: the low density lipoprotein receptor-related protein 1 (LRP1) and the receptor for advanced glycation end product (RAGE). A thickening of the epithelial basal membrane and greater collagen-IV deposition occurred around capillaries in CP, probably curtailing solute exchanges. Moreover, there was attenuated expression of epithelial aquaporin-1 and transthyretin (TTR) protein compared to Non-Tg mice. Collectively these findings indicate CP dysfunction hypothetically linked to increasing Aβ burden resulting in less efficient ion transport, concurrently with reduced production of CSF (less sink action on brain Aβ) and diminished secretion of TTR (less neuroprotection against cortical Aβ toxicity). The putative effects of a disabled CP-CSF system on CNS functions are discussed in the context of AD.

## Introduction

Alzheimer’s disease (AD) is age-related neurodegeneration characterized by plaques that consist mainly of amyloid-β (Aβ) peptides and neurofibrillary tangles (NFT) containing hyperphosphorylated tau protein. These protein distortions contribute to disrupted synapses and neurotransmission, progressive neuronal death, memory impairment, and cognitive disturbances (Church et al., 2014). *In vitro* and *in vivo* studies suggest that excessive Aβ peptides, at least in part, are causative or exacerbative agents in AD pathogenesis (Wang et al., 2006). Brain Aβ peptides of 40 (Aβ40) or 42 (Aβ42) amino acids are produced, with Aβ42 being more aggregation prone (Thinakaran and Koo, 2008).

Aβ peptides are generated from transmembrane amyloid precursor protein (APP) by β-secretase and γ-secretase in the endoplasmic reticulum, Golgi apparatus, and endosomal-lysosomal pathway (Selkoe, 2001; LaFerla et al., 2007). This Aβ-producing amyloidogenic pathway is active when APP has mutations at the cleavage sites by β- secretase or γ-secretase (Haass et al., 1995; Wolfe et al., 1999). In contrast, α-secretase cleaves APP within the Aβ domain, precluding the generation of Aβ in normal APP metabolism. While extracellular Aβ aggregation has long been considered as a key culprit in AD onset, intracellular Aβ accumulation is detected in neurons prior to the appearance of extracellular deposits (Wirths et al., 2001; Youmans et al., 2012) and is associated with cytotoxicity, dysfunction of organelles, and neurodegeneration (Bayer and Wirths, 2010).

While the rare autosomal dominant familial AD is mostly due to overproduction of Aβ (O’Brien and Wong, 2011) or enhancing Aβ protofibril formation, far more common is the late-onset sporadic AD (sAD), thought to be caused, in part, by decreased clearance of the Aβ peptide from the CNS (Dorfman et al., 2010; Mawuenyega et al., 2010; Silverberg et al., 2010a). Thus, the deregulation of Aβ clearance pathways in brain and at CP-CSF may be a central disease event in some AD cases.

Maintenance of equilibrium in volume and composition of CSF is vital for normal brain function, ensuring an optimal environment for neurons. Thus the choroid plexus (CP) part of the blood cerebrospinal fluid barrier (BCSFB) also mediates secretion of proteins and various processes that clear substances from the CSF, blood or vice versa (Balda and Matter, 1998; Al-Sarraf et al., 2007; González-Marrero et al., 2013). CP has traditionally been associated with active secretion of CSF into the cerebral ventricles. Secretion of CSF is a cardinal function of CP. In CNS, AQP-1, a water channel for high osmotic water permeability, is expressed mainly in the apical membrane of the ventricular CP (Boassa and Yool, 2005) and regulates formation of CSF. Noteworthy, >90% of the transthyretin (TTR) in CSF is synthesized within CP (Serot et al., 1997). TTR is the hallmark protein synthesized by CP; its level in the epithelium and CSF reflect the health of the BCSFB. The main carriers for Aβ transport across BBB from brain-to-blood, and blood-to-brain, are lipoprotein receptor-related protein (LRP1) and the receptor for advanced glycation end-products (RAGE), respectively. Aβ transporters in BBB endothelium are also found in CP epithelium, e.g., LRP1 (Pascale et al., 2011), megalin/LRP2, P-gp and RAGE (Hammad et al., 1997; Deane et al., 2003; Serot et al., 2003a; Crossgrove et al., 2005).

Continuous removal of Aβ species from CNS is important for preventing potentially neurotoxic accumulation in brain interstitial fluid (ISF) and CP. Since CP directly connects CSF with the ISF, the Aβ in the brain extracellular space can freely enter CSF and be transported at the BCSFB. Increasing data for animals support the notion that compromised function of CP and defective CSF production and turnover (Chiu et al., 2012), with diminished clearance of the Aβ peptides normally produced in brain, may be a mechanism implicated in the exacerbation of sAD (Silverberg et al., 2001, 2003; Serot et al., 2003b; Johanson et al., 2008; Wostyn et al., 2011).

Much understanding of how Aβ pathology develops has been driven by studies in animal models, which lacks proper reflection of the human disease, e.g., choroidal transport mechanisms and CSF homeostasis (Johanson et al., 2004; Spector and Johanson, 2013). Although there is no model capturing all the hallmarks of AD, it is possible to model Aβ deposition (albeit with cautionary limitations) when comparing to human metabolic and transport counterparts. The most popular animal models of AD are transgenic mice expressing human genes with mutations leading to familial AD.

Adult 3xTg-AD mice, harboring PS1/M146V, APPSwe and tauP301L human transgenes, is an animal model that mimics many critical hallmarks of AD (Oddo et al., 2003b). Adult 3xTg-AD displays cognitive deficits and other behavioral alterations at ages when overt neuropathology is not yet observed, although intraneuronal amyloid Aβ-peptide accumulation and synaptic (cholinergic) deficits are already being described at 6 months of age (Oddo et al., 2003b; Hedberg et al., 2010; Sterniczuk et al., 2010). Adult 3xTg-AD progressively develops Aβ plaques and NFT from 12 months, with temporal- and regional-specific profiles closely imitating their development in human AD (Oddo et al., 2003a,b). Currently, little is known about the spectrum of transporter and metabolic responses by CP to the disrupted CNS homeostasis in AD; or reciprocally, how CP malfunctions may contribute to (exacerbate) AD neuropathology.

We have sought to determine whether the pathological accumulation of CNS Aβ in 3xTg-AD mice have repercussions in the functional protein expression of CP. Moreover, because compromised metabolic functions of CP and defective CSF production (turnover) may be linked to worsening sAD, we decided to ascertain whether 3xTg-AD is useful to study the role of CP in AD.

## Materials and methods

### Animal model of AD

3xTg-AD mice harboring three mutant genes: beta-APP (APPswe), presenilin-1 (PS- 1M146V) and tauP301L, and the corresponding wild type mice were provided by Dr. Lydia Giménez-Llort (Autonomous University of Barcelona, Spain). Eight 3xTg-AD mice and eight non-transgenic control mice (Non-Tg), 16 month-old, were used. Tail DNA from WT and 3xTg-AD mice was genotyped to confirm the absence or presence of APP, PS1 and tau transgenes in Non-Tg and 3xTg-AD mice, respectively. Moreover the presence of senile plaques in hippocampus and cerebral cortex was confirmed by Congo red and thioflavin-S. Mice were kept at a constant temperature of 21 ± 2°C and 55 ± 8% relative humidity in light-dark 12–12 h. All experiments were conducted according to the European Directive 2010/69/EU for the maintenance and use of laboratory animals, which were approved by the Committee of Animal Use for Research at the University of La Laguna. The number of animals used, as well as stress and suffering of these subjects during handling and experimentation was minimized.

### Immunofluorescence

To study lateral ventricle CP, confocal microscopy was used. Brains were perfused transcardially by PBS 0.1 M followed by fixative (4% paraformaldehyde in PBS 0.1 M pH 7.4), embedded in paraffin and sectioned at 7 μm. After deparaffinization and rehydration, tissue sections were treated with 10 mM citrate buffer, pH 6, at 85°C for 20 min. Slides were treated 30 min with PBS containing 3% bovine serum albumin to block non-specific binding. After washing in PBS 0.1 M pH 7.4, sections were incubated overnight at 4°C with appropriate primary antibody: rabbit polyclonal anti- amyloid-β 1–40 (ABCAM, 1:500); rabbit polyclonal anti-amyloid-β 1–42 (Abbiotec, 1:200); rabbit polyclonal anti-low density LRP1 (Sigma-Aldrich, 1:200); rabbit polyclonal anti-receptor for advanced glycation end-products (Sigma-Aldrich, 1:400); rabbit monoclonal anti-aquaporin 1 (ABCAM, 1:200); rabbit polyclonal anti-transthyretin (Dako, 1:200). After washing, Cyanine 3 (Cy3) (red) goat anti-rabbit IgG conjugate antibodies (Invitrogen) were used as secondary antibodies for immunofluorescence. Nuclei were stained with 4’-6’ diamidino-2-phenylindole (DAPI). After 3 washes, samples were mounted in Vectashield Medium (Vector Laboratories Inc) for viewing by confocal microscopy (FV 1000 Olympus).

### Collagen-IV immunohistochemistry

CP collagen-IV was studied using light microscopy. Sections were treated as explained for confocal microscopy, but after hot citrate buffer they were washed with distilled water and quenched with 3% hydrogen peroxide for 10 min at room temperature to eliminate endogenous peroxidase activity. As a primary antibody, rabbit polyclonal anti-collagen type IV (ABCAM, 1:500) was used. Goat anti-rabbit IgG conjugate antibodies (Invitrogen) were used as secondary antibodies. In light microscopy, the DAKO StreptABC complex/HRP Duet, Mouse/Rabbit procedure was used and the peroxidase reaction product visualized with diaminobenzidine.

### Quantification of immunostaining

From each animal/group we obtained near 60 slices of CP of lateral ventricle. To study each antibody we used 7–8 slices. The intensity of immunostaining was measured in whole CP present in the slice. Fluorescence intensities from images were semi-quantitatively analyzed by densitometry. Immunohistochemistry slides were converted to digital images by using an LEICA DMRB photomicroscope with an LEICA DC 300 F camera (Germany) as 8-bit acquisitions of color. Image analysis was completed in Image J (v. 1.43 u, NIH, Bethesda, MD, USA). The “Mean Gray Value” was measured for all stained tissue. This value gives the average stain intensity in gray scale units for all threshold pixels.

### Statistical analysis

A one-way ANOVA was used for data comparison between 3xTg-AD and Non-Tg mice. ANOVA was conducted with IBM SPSS statistic 19 software where data were considered as statistically significant at *p* < 0.01.

## Results

### Amyloid-beta distribution in CP of 3xTg-AD and Non-Tg mice

**Figure 1:**
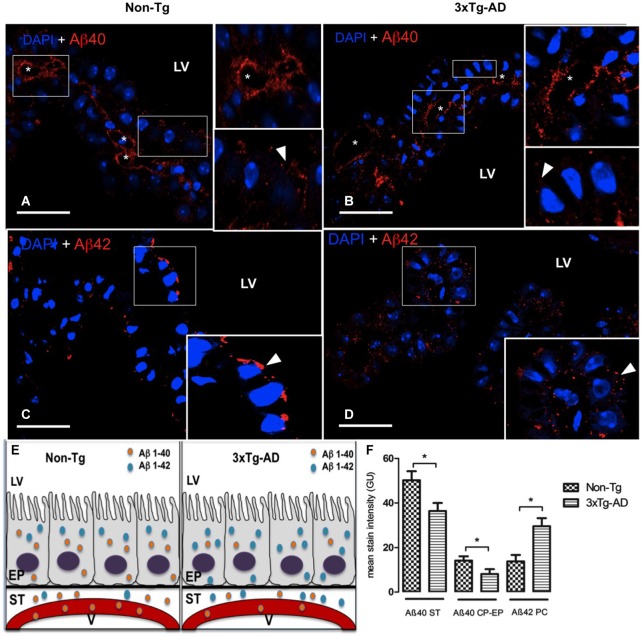
**Choroid plexus (CP) confocal microscopy images immunolabeled from Non-Tg (A,C) and 3xTg-AD mice (B,D) stained with anti-Aβ40 (A,B) and anti-Aβ42 (C,D)**. The asterisks show the blood vessels staining and arrowheads show the cytoplasmic staining of CP epithelial cells (CP-EP) of lateral ventricle (LV). According to figure (see also the results in text) in the left bottom **(E)** is also shown a scheme about the distribution of Aβ40 and Aβ42 in vessels (V), stroma (ST) and epithelial cells (EP) of 3xTg-AD compared to Non-Tg mice. On the right bottom **(F)** are presented values of stain intensitites for Aβ40 and Aβ42 as the means ± SEM; *n* = eight animals per group; * *p* < 0.01 3xTg-AD *vs*. Non-Tg mice. Scale bars, 30 μm.

Aβ is a short peptide generated from APP with two main breakdown products, Aβ40 and Aβ42. Here we examined these peptides in different compartments related to CP in 3xTg-AD and Non-Tg mice. Also is included a schematic summary of their distribution in lateral ventricles, epithelial cells, stroma and blood vessels (Figure 1E).

Confocal microscopy images revealed a strong staining of stroma and vessels of CP and a moderate cytosolic signal with anti-Aβ40 (Figures 1A,B). We found reduced Aβ40 expression in cytosol, stroma and vessels of CP of 3xTg-AD *vs*. Non-Tg mice. The difference observed in Aβ40 staining was greater in the stroma surrounding blood vessels than in CP epithelium. A reduced Aβ40 expression was detected in CP stroma surrounding blood vessels and in CP epithelium from 3xTg-AD *vs*. Non-Tg mice (Figure 1F; * *p* < 0.01 3xTg-AD *vs*. Non-Tg mice).

Regarding Aβ42, staining revealed differences in signal strength and distribution. Non-Tg mice presented a CP epithelium with Aβ42 mainly cytoplasmic and specially in areas near the apical pole (Figure 1C); in 3xTg-AD mice, Aβ42 distributed uniformly through out the cytosol (Figure 1D). Is also important to point out that Aβ42 had a more than a two-fold increased expression in stroma of 3xTg-AD mice compared to the weak expression in Non-Tg mice (Figure 1F; * *p* < 0.01 3xTg-AD *vs*. Non-Tg mice). Also, vessels lacked Aβ42 staining in Non-Tg animals (Figure 1C).

### LRP1 and RAGE expression in CP epithelium

**Figure 2:**
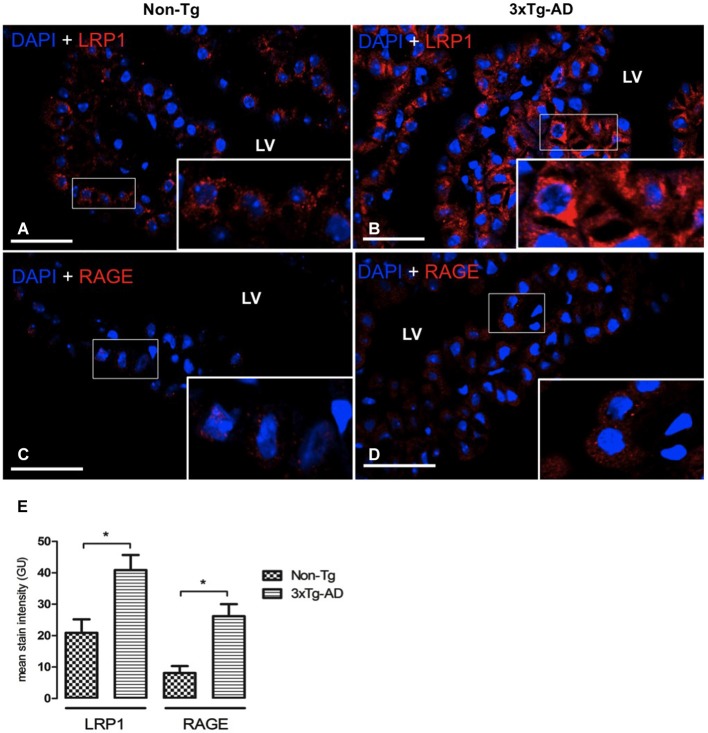
**CP confocal microscopy images immunolabeled from Non-Tg (A,C) and 3xTg-AD mice (B,D) stained with anti-LRP1 (A,B) and anti-RAGE (C,D)**. An increase in cytoplasmic expression of LRP1 and RAGE in epithelial cells of lateral ventricle (LV) of 3xTg-AD compared to Non-Tg mice is shown. On the left bottom **(E)** are represented values of stain intensitites for LRP1 and RAGE as the means ± SEM; *n* = eight animals per group; * *p* < 0.01 3xTg-AD *vs*. Non-Tg mice. Scale bars, 30 μm.

LRP1 and RAGE are the main carriers for Aβ transport across BBB from brain-to-blood, and blood-to-brain, respectively. Therefore, the next step was to analyze expression of these transporters in CP of the 3xTg-AD and Non-Tg mice. LRP1 was expressed on cytoplasm of epithelial cells in Non-Tg animals (Figure 2A) and in 3xTg-AD mice (Figure 2B). Cytosolic LRP1 protein presented a two-fold increase in 3xTg-AD compared Non-Tg (Figure 2E). Confocal microscopy images for RAGE in CP from Non-Tg and 3xTg-AD mice are also shown (Figures 2C,D). The location of RAGE staining in both groups of animals was only cytosolic and lacked detection either in CP vessels or basement membrane (Figures 2C,D). We found ~four-fold cytoplasmic increase in RAGE expression in epithelial cells of 3xTg-AD *vs*. Non-Tg (Figure 2E; * *p* < 0.01 3xTg-AD *vs*. Non- Tg mice).

### Collagen-IV deposition in CP basement membrane

**Figure 3:**
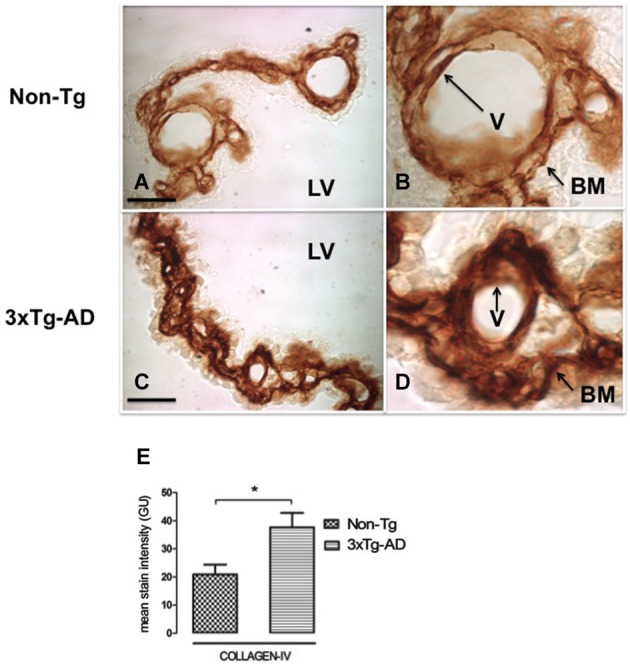
**CP immunohistochemistry images from Non-Tg (A,B) and 3xTg-AD mice (C,D) show CP basement membrane and basal membrane of CP vessels (V) immunostained with anti-collagen-IV (arrows)**. **(B,D)** are enlarged images of **(A,C)** respectively. An increased collagen-IV expression in CP of 3xTg-AD **(C,D)** compared to Non-Tg mice **(A,B)** is shown. On the left bottom are represented values of stain intensities for collagen-IV **(E)** as the means ± SEM; LV: lateral ventricle; *n* = eight animals per group; * *p* < 0.01 3xTg-AD *vs*. Non-Tg mice. Scale bars, 60 μm.

CP is a highly vascularized structure receiving 5–10 times more blood flow than other brain regions (Szmydynger-Chodobska et al., 1994). This physiologic state of brisk blood flow would make CP more sensitive to reduced vascular perfusion. In order to link possible changes in CBF with the state of both of the vessels and basement membrane, we also examined collagen-IV expression. Figure 3 presents CP staining with anti-collagen type IV in Non-Tg (Figures 3A,B) and 3xTg-AD mice (Figures 3C,D). Collagen-IV is widely located only in CP epithelial cells basement membrane and the blood vessel basement membrane, a collagen-IV cell staining lacked detection in whole CP. Collagen-IV labeling was ~two-fold higher in 3xTg-AD in comparison with Non-Tg mice (Figure 3E; * *p* < 0.01 3xTg-AD *vs*. Non- Tg mice).

### AQP-1 and TTR expression in CP

**Figure 4:**
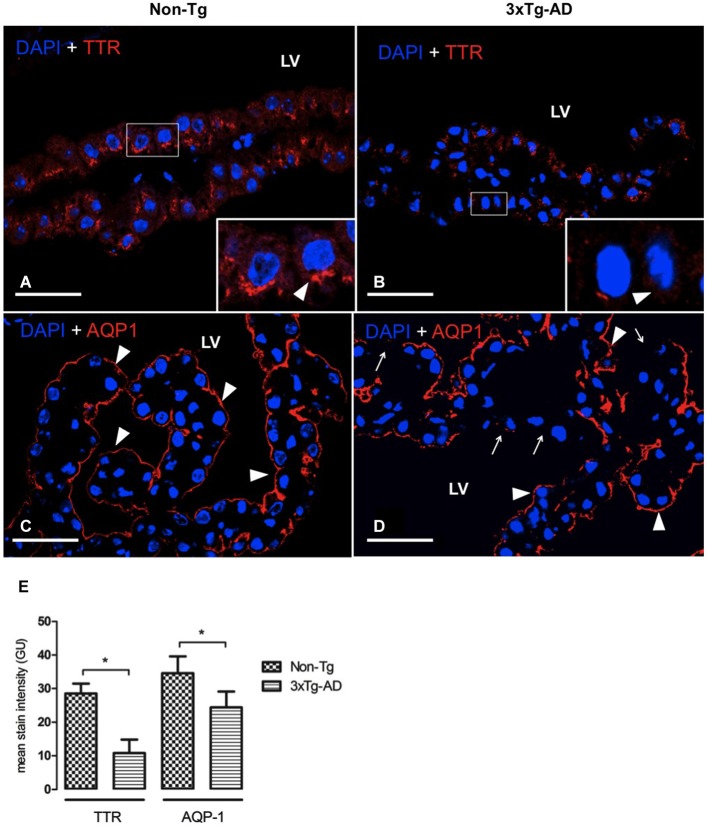
**CP confocal images immunolabeled from Non-Tg (A,C) and 3xTg-AD mice (B,D) stained with anti-TTR (A,B) and anti-AQP-1 (C,D)**. A reduction in cytoplasmic expression of TTR **(A,B)** as well as in AQP-1 expression in the apical membrane of epithelial cells **(C,D)** is shown in 3xTg-AD when compared to Non-Tg mice (arrowheads). The arrows in **(D)** indicate areas where AQP1 was not detected on the apical membrane of CP epithelium. On the left bottom side are represented values of stain intensitites for TTR and AQP-1 **(E)** as the means ± SEM; LV: lateral ventricle; *n* = eight animals per group; * *p* < 0.01 3xTg-AD *vs*. Non- Tg mice. Scale bars, 30 μm.

In AD, the adverse effects of CSF stasis (decreased turnover rate) are potentiated by declining ability of CSF to inhibit formation of Aβ oligomers (Serot et al., 2003b, 2012; Silverberg et al., 2003; Wostyn et al., 2011). We assessed expression of two proteins in CP secretory functions. These are TTR, one of the major proteins synthetized by CP, and AQP-1, a water channel transport protein involved in CSF production. TTR was located in cytoplasm of CP epithelial cells as in 3xTg-AD as in Non-Tg mice. TTR present in CP was significantly reduced in 3xTg-AD compared to Non-Tg mice (Figures 4A,B). This expression was >3 fold lower in 3xTg-AD than Non-Tg (Figure 4E; * *p* < 0.01 3xTg-AD *vs*. Non- Tg mice).

AQP-1 expression was located on the apical membrane of CP epithelial cells in Non-Tg (Figure 4C) and 3xTg-AD mice (Figure 4D). While in Non-Tg mice AQP-1 staining was distributed in whole apical pole of CP epithelial cells, we observed that some epithelial cells showed a lack of AQP-1 expression on their apical membranes. The values of AQP-1 staining were 31% lower in 3xTg-AD compared to Non-Tg mice (Figure 4E).

## Discussion

3xTg-AD mice at 16 months exhibits age- related pathology similar to human AD (Oddo et al., 2003b). We analyzed: (i) the amount of Aβ fragment material in CP as well as the transporters that move Aβ into and out of CP, (ii) the expression of collagen at transport interfaces within the BCSFB; and (iii) the effects of highly-expressed human AD-linked transgenes on the synthesis of CP functional proteins such as TTR and AQP-1.

### Choroid epithelial cell Aβ

Aβ accumulates in CP of AD patients. Similarly, we found increased Aβ42 in epithelial cytoplasm of 3xTg-AD mice. Choroid cell Aβ is also elevated in aging rats, various Tg mouse models of Aβ, and in Pb-treated mice. Changes in CP Aβ levels reflect altered homeostatic transport across the BCSFB and/or greater local synthesis of Aβ in choroid cells. Excess Aβ in CP probably alters homeostatic functions of the BCSFB (e.g., increased barrier permeability) or compromises organic molecule transport adjustments to protect the CSF-brain interstitial environment. Toxic levels of Aβ in CP could potentially alter enzymatic reactions that carry out homeostatic functions such as forming CSF. Therefore, attention needs to be paid to Aβ loading (burden) in CP as well as brain.

### Aβ transporters in CP

In the 3xTg-AD mice, we found increased LRP1 and RAGE in CP cytosol. AQP-1 moves between cytosol and external limiting membranes (e.g., apical) of CP to control functional activity (Kalani et al., 2012); also, LRP1 and RAGE in cytosol probably inserts into cellular membranes -also needing elucidation is how cytosolic transport proteins insert into organelles to control Aβ metabolism and trafficking within the cell-. Clearly, substantial Aβ data for BBB indicate that expression levels of endothelial LRP1, LRP2, P-gP and RAGE markedly affect Aβ burden in cortex. Similar transport/distribution principles apply to BCSFB, in regard to clearing excess Aβ out of CNS.

There is increased transcription of the Aβ efflux membrane transporters, LRP1 and P-gp, no change in RAGE expression and a decrease in Megalin/LRP2 at the BCSFB during normal *aging* in rodents (Pascale et al., 2011). Although aging CP transport phenomena mimics those of AD, they may differ in degree of affectation. Age- dependent alterations in CP Aβ transporters were associated with decreased Aβ42 in CP, and were reciprocal to corresponding changes in LRP1 and PgP transporter expression at the aging BBB (Silverberg et al., 2010a,b). This suggests compensatory clearance via BCSFB of Aβ in aging, even as the BBB LRP1 and P-gp transporters are failing (Pascale et al., 2011).

Contrary to non-diseased aging, we found in 3xTg-AD mice elevated Aβ42 and increased expression of cytosolic LRP1 in CP epithelium (Figures 2B,E). Popular animal models of AD, including 3xTg-AD, are mice overexpressing human genes with mutations that cause familial AD. These Tg mice overaccumulate Aβ in CP and brain. Aβ42 is more problematic because it aggregates and promotes deposits earlier in disease than Aβ40 (Iwatsubo et al., 1994). Thus, abundant in brain are Aβ40 and Aβ2, the first being the prevalent fragment, and the latter more amyloidogenic (Abramowski et al., 2012).

### Aβ uptake from CSF by CP

CP is contiguous with CSF and cerebral interstitial fluid (ISF; Johanson, 2003). Thus, Aβ in brain ISF freely enters CSF (Gu et al., 2014) and flows/diffuses to nearby CP for reabsorption into choroidal venous blood. Moreover, interstitial Aβ drainage from brain to ventricles seems to be the major origin of Aβ in CSF because changes in CSF Aβ levels reflect changes in brain Aβ following therapeutic treatments (Lu et al., 2013). CP epithelium is exposed to several-fold higher Aβ40 concentrations in CSF than that of Aβ42 (Cirrito et al., 2003; Head et al., 2010; Borghys et al., 2014; Savage et al., 2014).

LRP1 in CP has a major role in removing Aβ from CSF via clearance transport at the apical membrane. Why does Aβ42 accumulate in cytosol near the apical membrane? Upon transport into the choroid cell from CSF, there is probably epithelial metabolism of cytoplasmic Aβ40 (Crossgrove et al., 2007) and/or efflux at the choroid cell-ISF interface into venous blood (Crossgrove et al., 2005). More needs to be known about efflux transport in the basolateral membrane because this is the final step in disposing CNS-CSF Aβ into blood, thus protecting CP epithelium and ultimately the brain from Aβ toxicity. In acute Pb-toxicity model, the decreased expression of LRP1 in CP epithelium was associated, paradoxically, with increased Aβ in CP (Behl et al., 2010); however, CP Aβ efflux into blood was uncharacterized; a factor also affecting epithelial Aβ concentration.

### Metabolic processing of CP Aβ

Following uptake of Aβ from outside CP, or its enzymatic generation within the epithelium, there is processing of the amyloid peptides to minimize toxicity. Low solubility and elevated Aβ42 in CP may exceed lysosomal degradation capacity. Notably, intact lysosomes are more prevalent in fluorescein- labeled Aβ0 (F-Aβ40) treated cells than in F-Aβ42 treated cells, likely due to less F-Aβ40 inability to accumulate in lysosomes (Omtri et al., 2012). Cell culture reveals substantial intracellular Aβ and comparably little secretion, particularly of Aβ42 (Maruyama et al., 1995). Moreover, continuous uptake data suggest that, over time, Aβ42 might escape degradation and start to accumulate (Fuentealba et al., 2010). All in all, CP Aβ accumulation is a function of Aβ metabolism as well as uptake from CSF.

Our current study shows significantly reduced Aβ40 in CP epithelium and stroma in 3xTg-AD *vs*. Non-Tg mice (Figures 1A,B,F). Aβ40 impacts lysosomal integrity less because of smaller propensity to aggregate and greater susceptibility to lysosomal proteases. Aβ40 clears rapidly after i.c.v. injection (Ghersi-Egea et al., 1996; Ji et al., 2001), and may also be degraded faster in CP. Interestingly, in Tg mouse lines generating Aβ40 (APP47) and Aβ42 (APP48) in neurons, APP47 mice only show somatic Aβ granules consistent with a more rapid, complete degradation of Aβ40 (Omtri et al., 2012).

Our findings provide evidences CP internalize Aβ40 and Aβ42 via different mechanisms. Such regulated trafficking along distinct pathways suggests that Aβ40 and Aβ42 exercise differential effects on CP epithelial cells. Further studies should investigate the intracellular pharmacokinetics of Aβ40 and Aβ42 following uptake at the BCSFB, and evaluate the tendency of Aβ fragments to accumulate in cellular compartments susceptible to Aβ toxicity.

### Do plasma increased Aβ and soluble LRP1 also affect CP-CSF Aβ?

Aβ may enter CP from plasma as well as CSF. We show increased choroid cell Aβ42 immunofluorescence, punctuated in CP of 3xTg-AD compared with Non-Tg mice (Figures 1C,D,F). In neurologically-healthy humans and mice, a soluble LRP1 species, sLRP1, binds >70% of vascular-circulating Aβ; this prevents free Aβ access to brain (and presumably CP epithelium; Sagare et al., 2007). In AD patients and Tg mice, Aβ binding to sLRP1 is severely compromised. This results in elevated levels of free Aβ40 and Aβ42 in plasma (Sagare et al., 2011) that (in addition to LRP1-mediated Aβ efflux into blood) can *re-enter* brain via RAGE transport across BBB, and possibly CP (Deane et al., 2003; Donahue et al., 2006; Sagare et al., 2007).

RAGE transports Aβ into CNS from plasma, where it may be elevated in AD (Deane et al., 2003). RAGE expression increases in neurons, astrocytes and cerebrovascular endothelial cells in an Aβ-rich environment, seen in AD models and dementia (Deane et al., 2003; Choi et al., 2014). Such influx, if accelerated by elevated plasma Aβ via upregulated RAGE in BBB and BCFSB, would amplify the Aβ-mediated pathogenic/metabolic responses. Our study does show increased cytosolic RAGE expression in CP epithelial cells and an Aβ42 increase in CP stroma of 3xTg-AD *vs*. Non-Tg mice (Figures 2D,E). Similarly to BBB, Aβ42 accumulation in CP blood and stroma may induce a RAGE increase, which could lead to a significant Aβ increase in CP.

### Altered basement membranes in the BCSFB and BBB of 3xTg-AD mice

The basement membrane (Deane et al., 2004) thickens in the BBB of 3xTg-AD mice; this thickening can modulate hemodynamic responses, microvessel integrity/permeability and molecular fluxes via transporter expression/activity in the barrier. Animal models reveal mechanisms that link reduced cerebral blood flow (CBF) to capillary state. Permanently ligated common carotid arteries in rats model chronic cerebral hypoperfusion, when CBF is reduced to ~70% (Tsuchiya et al., 1992). This coincides with falling CBF in the hippocampus and temporal cortex in AD (Eberling et al., 1992; Ohnishi et al., 1995; Tang et al., 2012; Mattsson et al., 2014). A previous 3xTg-AD study described reduced vascular volume in brain of 11-month-old mice; BBB functional integrity was maintained even though reduced vascular volume was accompanied by accumulated collagen I/IV and a thickened vascular basement membrane (BM; Bourasset et al., 2009). Microvascular BM thickening, with more collagen-IV, has been also shown in human AD (Kalaria and Pax, 1995; Claudio, 1996); pathophysiologic analyses of transporters and fluxes in thickened BBB membranes need to be done.

CP is highly perfused, much greater than brain. Any dwindling of this brisk choroidal blood flow, as in AD, might impact endothelial cells and their underlying BM. In the current study, the apparent thickness of BM in CP was assessed immunohistochemically by measuring collagen-IV (Farkas and Luiten, 2001). For the first time, we show significantly accumulated collagen-IV in BM of CP in 3xTg-AD mice (Figures 3C–E). The exact reason for increased collagen-IV and subsequent thickening of the BMs in AD, at *both* the capillary and epithelial BMs, needs full characterization. According to Fick’s diffusion theory, a major factor affecting diffusion across biological membranes is membrane thickness. BM thickening reduces permeability (Zwolinski et al., 1948) and makes plasma ultrafiltration, CP epithelial oxygenation and CSF formation all less efficient. This idea fits our findings of lower expression of AQP-1 and TTR in CP in 3xTg-AD mice.

### CSF bulk flow attenuation: reduced excretion of CSF Aβ

Along with reduced clearance of Aβ across BBB with increasing age and AD, there is also a decline in Aβ clearance from CNS via CSF bulk flow into the arachnoidal-venous system (Silverberg et al., 2010b; Pascale et al., 2011; Serot et al., 2011). What mechanisms underlie this phenomenon? CSF formation by CP, and its eventual turnover into the ventriculo-subarachnoid CSF system, depends upon the molecular integrity of CSF- transport enzymes and AQP-1; the expression of both are reduced in 3xTg-AD mice, probably by toxic levels of Aβ in CP. Our working model supports the notion that accumulating Aβ42 in choroidal epithelium contributes to a dysfunctional BCSFB. In fact, a sequence of pathological steps that link Aβ accumulation with an AD progression-related increase in oxidative damage, has recently been proposed (Krzyzanowska and Carro, 2012).

The necessarily high water permeability of the CP interface is made possible, in part, by AQP-1, a water channel expressed strongly at the ventricular- facing surface (Boassa and Yool, 2005). Senescent rats are less able to form CSF (Masseguin et al., 2005); a reduced AQP-1 expression in CP accompanies slower rates of CSF secretion in aging (Redzic et al., 2005). Similarly, reduced CSF secretion occurs in aging and AD (Johanson et al., 2008; Chen et al., 2009; Serot et al., 2012). The reduced AQP-1 expression in CP of 3xTg-AD mice (Figures 4C–E) probably impairs CP capacity to secrete CSF; consequently, the slowing of CSF convection would reduce Aβ removal from 3xTg-AD mice CNS via ventricular bulk flow.

### Lower expression of CP transthyretin: implications for reduced Aβ removal

Decreased CP protein synthesis/secretion as well as slower CSF renewal accelerate the progressing neuropathology. As mentioned, some CP proteins interact neuroprotectively with Aβ and this could be the basis for altered Aβ clearance; lower TTR in CSF would mean less neuroprotection afforded via enzymatic degradation of Aβ and tetramer stabilization binding. TTR is a major protein synthesized and secreted by CP into CSF (Dickson et al., 1986); this tetrameric protein binds to and stabilizes soluble Aβ (Tang et al., 2004). We found reduced TTR expression in CP of 3xTg-AD *vs*. Non-Tg mice (Figures 4A,B,E). Lower TTR expression in CP of 3xTg-AD mice likely leads to deficiency of TTR in CSF. Decreased CSF levels of TTR occur in severe dementia and AD (Riisøen, 1988; Serot et al., 1997). Since CP is the primary source of TTR in the CNS, a CSF-deficient TTR presumably results from decreased CP synthesis/secretion in aging and AD (Stoquart-ElSankari et al., 2007). Diminishing CNS TTR has been linked to cognitive decline with age (Preston et al., 2005). A key question is whether excess Aβ in CP directly down-regulates TTR expression in epithelial cells.

## Conclusions

Our findings prompt consideration of identifying ways to protect or restore the gradually-failing functions of the BCSFB in the face of metabolic dysfunctions in the aging, diseased CNS. A healthy CP in advanced life stages would confer numerous benefits on the brain. Moreover, these results may be considered for futures pharmacological treatments in order to restore impaired functions of CP in AD.

## Conflict of interest statement

The authors declare that the research was conducted in the absence of any commercial or financial relationships that could be construed as a potential conflict of interest.
